# Case Report: Endoscopic-assisted cistern ostomy with Ommaya reservoir insertion for trapped temporal horn: surgical technique and case illustration

**DOI:** 10.3389/fsurg.2026.1621466

**Published:** 2026-08-06

**Authors:** Sultan AlSaiari, Bassam A. Fallatah, Shahid Bashir, Essam M. Rezk, Rami Alhazmi

**Affiliations:** 1 Department of Neurosurgery, Neuroscience Center, King Fahad Specialist Hospital- Dammam, Dammam, Saudi Arabia; 2 Research Center, King Fahad Specialist Hospital-Dammam, Dammam, Saudi Arabia; 3 Department of Neurosurgery, King Abdullah Medical City, Makkah, Saudi Arabia; 4 Department of Neurosurgery, Faculty of Medicine, Tanta University, Egypt; 5 Department of Radiology, King Fahad Specialist Hospital-Dammam, Dammam, Saudi Arabia

**Keywords:** cisternostomy, intraventricular, morbidity, myxoid tumor, trapped ventricle

## Abstract

**Background:**

Neuro-endoscopy offers solutions to several challenges encountered during intraventricular tumor surgery. In this case, technical nuances included performing a ventriculo-subarachnoid cisternostomy for cerebrospinal fluid (CSF) diversion, and the postoperative application of a tightly stitched dressing gauze secured with silk sutures for a few weeks to minimize the development of CSF collections (pseudomeningocele) beneath the skin. Such collections can exhibit high protein content due to slow absorption, complicating patient management.

**Case presentation:**

We report the case of a 25-year-old male who presented with bilateral visual loss leading to blindness, secondary to an intraventricular tumor with longstanding hydrocephalus. He initially underwent a biopsy at another healthcare facility and was diagnosed with papilloma. The patient was subsequently referred to our center for a re-attempt at gross total resection via a standard temporal craniotomy, followed by histopathological evaluation. The final pathology revealed a myxoid variant with angiomatous features, confirmed by an internationally recognized reference laboratory. Following tumor resection, a trapped temporal horn developed due to the intraventricular location of the lesion. This was managed using an endoscopic approach aimed at diverting the cyst to the basal cistern through fenestration under intraoperative image-guidance (S7 Navigation, AxiEM system). A 2.7 mm rigid endoscope (Karl Storz) was employed to establish communication between the trapped ventricle and the subarachnoid space via fenestration toward the basal cisterns. An Ommaya reservoir was subsequently placed to prevent reaccumulation. These steps are described in detail in the surgical technique section.

**Discussion:**

Endoscopic ventriculo-cisternostomy is a safe and effective procedure for the treatment of symptomatic temporal horn entrapment in carefully selected cases. However, it carries risks of morbidity and complications in inexperienced hands and requires further validation before being established as a standard approach for this condition.

## Introduction

Surgical resection of intraventricular tumors remains a significant challenge in neurosurgical practice due to their deep-seated location and proximity to critical neurovascular structures, necessitating an extremely meticulous approach (1). Although various techniques have been described for tumors in this region, microsurgery remains the gold standard for intraventricular tumor management despite its limitations. Technological advancements have led to the increased use of neuro-endoscopy as a minimally invasive alternative aimed at managing intraventricular pathology (2). Intraventricular temporal horn tumors demonstrate considerable variability based on tumor type, completeness of resection, and response to adjuvant therapies. Generally, tumors with benign histology and complete surgical resection are associated with a better prognosis, while malignant or incompletely resected tumors tend to have poorer outcomes (3).

Neuro-endoscopy addresses some of the challenges encountered in intraventricular tumor surgery by improving illumination and visualization of anatomically difficult-to-access regions. Endoscopic techniques minimize the need for extensive tissue dissection and retraction, which are often required in conventional microsurgical approaches (4). The history of neuro-endoscopy dates to the early 1920s, when Walter Dandy introduced “cerebral ventriculoscopy” as a technique for visualizing the ventricular system and managing hydrocephalus (5, 6).

Myxoid mesenchymal intracranial tumors are rare and pose significant classification challenges due to their infrequency (7). Several case reports have documented intracranial mesenchymal tumors with myxoid variants, often associated with FET-CREB gene fusions. For instance, a 2017 study reported four cases of intracranial myxoid mesenchymal neoplasms in children and young adults, identifying fusions such as EWSR1-CREB1, EWSR1-CREM, or EWSR1-ATF1 (8).

Given the rarity of these tumors, their clinic pathological features, treatment strategies, and prognosis remain largely undefined (8). This paper aims to describe the safety and feasibility of a novel surgical technique ventriculo-subarachnoid fenestration for the treatment of trapped temporal horn secondary to an intraventricular cystic tumor. The technique utilizes neuro-endoscopy to create a shunt into the subarachnoid space, offering a minimally invasive approach to treat the associated hydrocephalus.

## Case presentation

**Figure 1:**
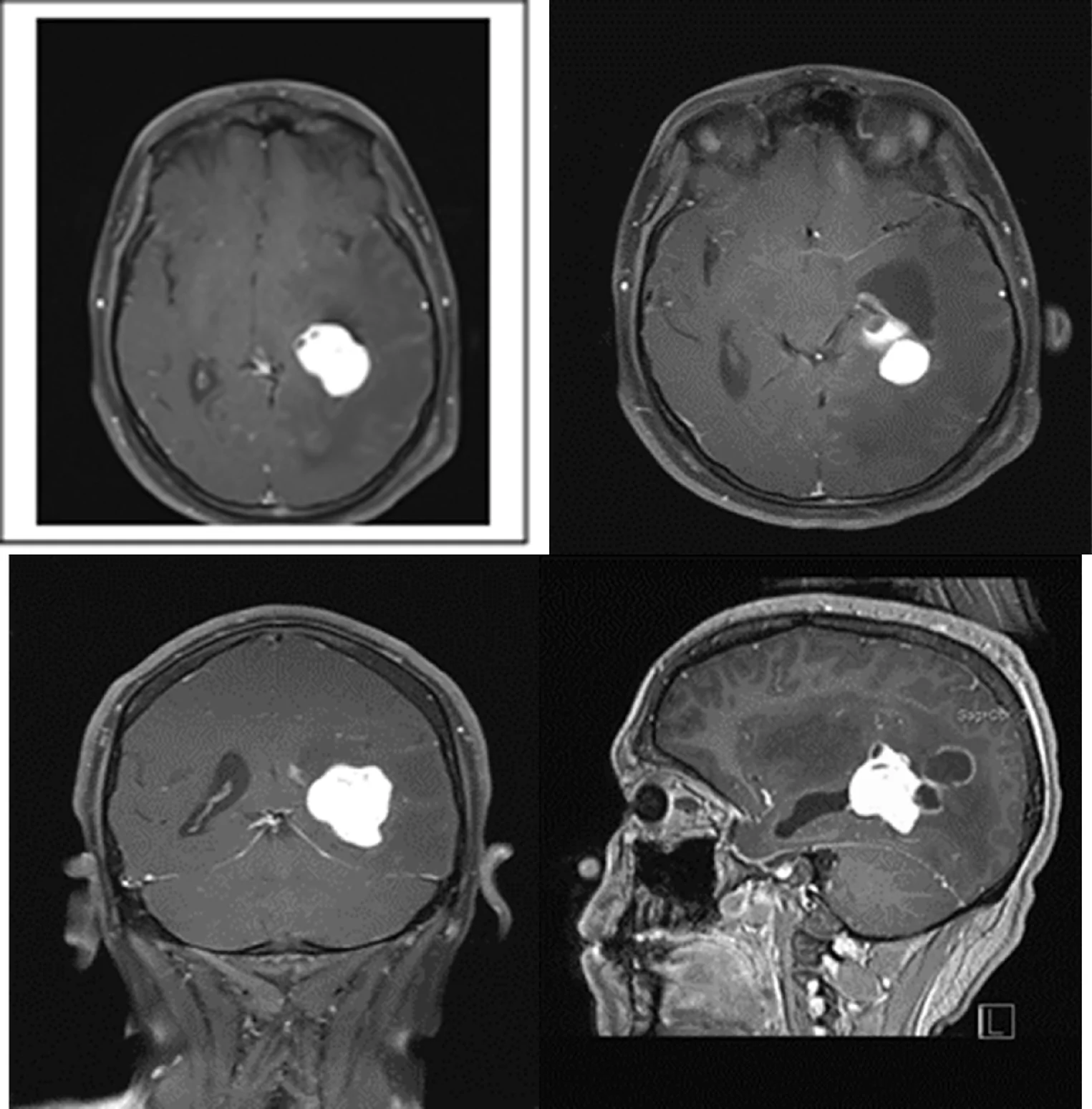
Preoperative: MRI axial, coronal, and sagittal with gadolinium contrast demonstrating left trigon of temporal horn intraventricular enhancing lobulated solid with cystic mass causing entrapment with hydrocephalous due to disconnection of temporal horn from the rest of ventricular system.

We report a rare case of an intraventricular tumor in a 25-year-old male who has no significant medical history. The patient presented with progressive bilateral vision loss over the course of several weeks and initially sought medical attention at another facility. Magnetic resonance imaging (MRI) of the brain revealed a lobulated intraventricular lesion (Figure 1). He subsequently underwent a craniotomy for tumor biopsy, and histopathological examination confirmed the diagnosis of an intraventricular papilloma.

Afterward the patient was referred to King Fahd Specialist Hospital-Dammam in order to deal with such tumor. At the time of presentation, he had been experiencing elevated intracranial pressure (ICP), progressive vision loss, and seizures despite being on antiepileptic medications. On examination, he was conscious, alert, and oriented. Cranial nerve assessment was unremarkable except for bilateral blindness. Motor function was intact, with no sensory deficits. Fundoscopic examination revealed bilateral optic atrophy.

The diagnosis of a myxoid variety with angiomatous characteristics was verified when gross complete resection via standard temporal craniotomy was accomplished. The surgical course was complicated by temporal horn hemorrhage and subsequent cystic dilatation of the confined ventricle hampered the surgery in this case, requiring numerous EVD insertions for over eight weeks and unsuccessful VPS insertions in between. The rationale of intraventricular hemorrhage could explained which might be related to the location of the tumor, injury of the choroid plexus, or residual blood along the surgical cavity through migrating to the ventricular system. The primary surgeon transferred the case to our care after all prior attempts and the CSF leak's persistence, and we chose to install a Ventriculo-cisternal Ommaya reservoir.

As shown in (Supplementary Figure S1), as symptomatic cystic dilatation continues, decision has been made for inserting Omaya reservoir connecting the dilated cyst to basal cistern by creating adjuvants holes that communicates the cyst to mesencephalic cistern allowing free drainage, which showed resolution of the cystic dilatation as shown in (Supplementary Figure S2A “post op).

**Figure 2:**
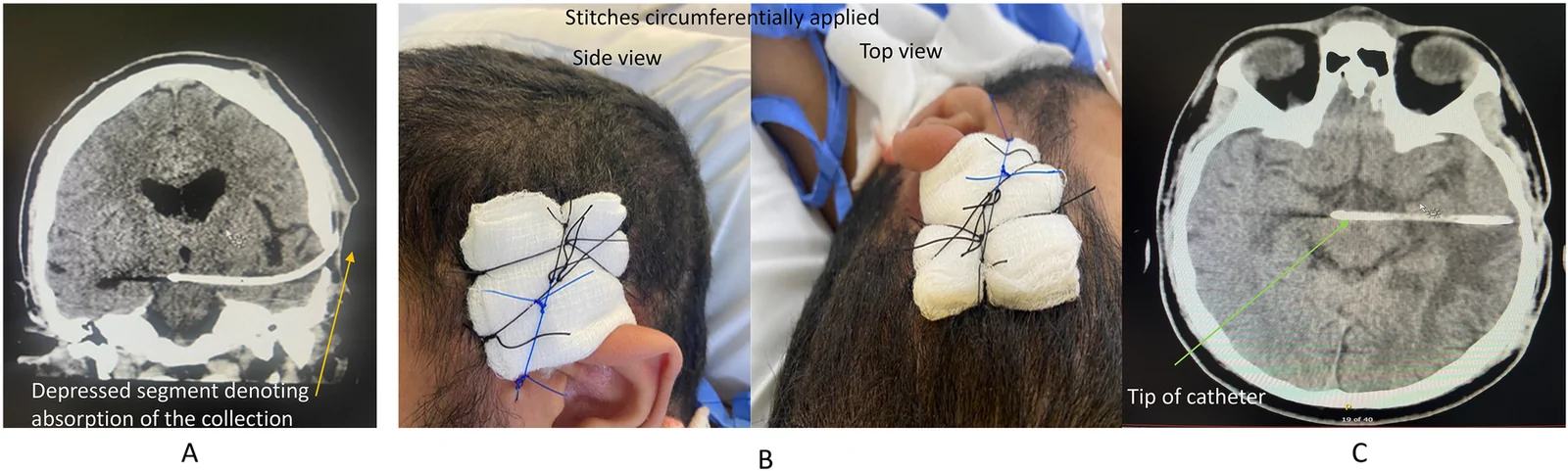
**(A)** CT Scan coronal view of the brain following gauze application technique showing subside of the collection with significant improvement in comparison to the pre op scan. Arrow (yellow) pointed toward the depression due to fixed applied gauze. **(B)** Images demonstrating the application of stitched dressing gauze circumferentially over the wound, tight by silk and proline stitches at the corners in both directions, craniocaudal and mediolateral, with a sealing effect. **(C)** CT Scan axial view of the brain four years follow-up showing subside of the collection with no recurrence and tip of catheter remain in place (green arrow).

The patient had temporary period of improvement in his clinical condition for few weeks, then he had persistence headache and then developed seizure with repeating imaging showed re-expansion of the cystic temporal horn which was caused by migration of the catheter holes to the basal cistern and the re-expansion of the cyst starts again as shown (Supplementary Figure S2B” post op). Later he underwent revision surgery with re-creation of multiple holes along the Omaya catheter which was secured by a stich at the burr hole as described in the surgical technique section. Follow-up scan revealed presence of the subcutaneous collection at the exit site but no recurrence of the cyst as shown in (Figure 2A). CT scan following gauze application technique showing subside of the collection with significant improvement in comparison to the pre op scan as shown in (Figure 2C). The other challenge facing was to address the collection with pseudo meningocele by using technique of application stitched dressing gauze circumferentially over the wound, tight by silk and proline stiches at the corners in both directions craniocaudal and mediolateral with sealing effect, as shown in (Figure 2B).

## Surgical technique

**Figure 3:**
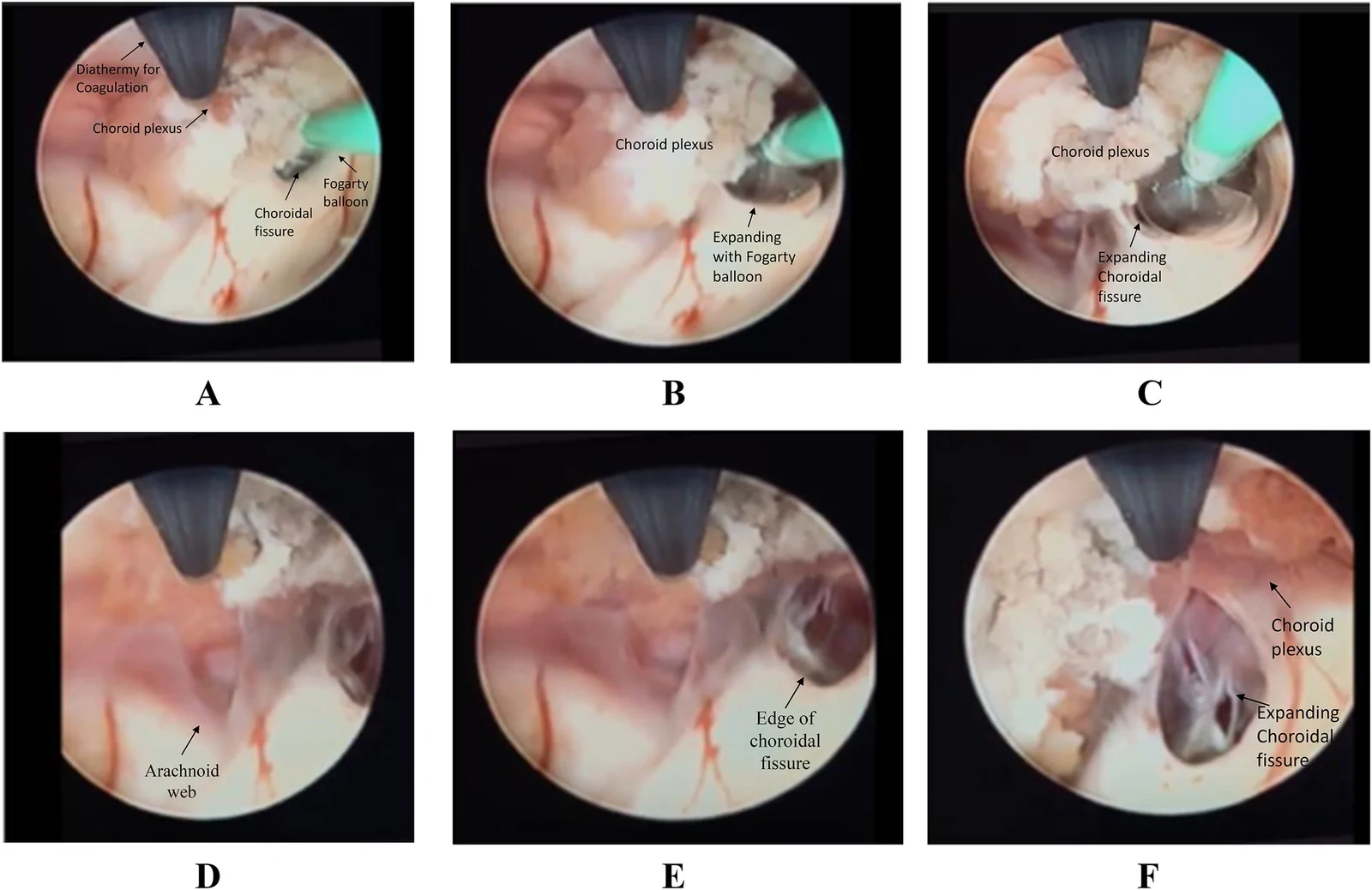
Illustration of the operative technique demonstrating the surgical steps for creating a cisternostomy through the temporal horn of the lateral ventricle, establishing a ventricular-subarachnoid shunt using a Fogarty catheter. **(A)** Coagulation of choroid plexus. **(B)** Deploying Fogarty balloon. **(C)** Expanding the choroidal fissure. **(D)** Releasing the membrane adhesions & coagulation of arachnoid web. **(E)** Fenestration of arachnoid web. **(F)** Opening of the choroidal fissure guiding to the basal cistern.

The surgical approach in our case utilized intraoperative image-guidance tools (S7 Navigation, AxiEM system). A 2.7 mm rigid endoscope (Karl Storz) was employed to connect the trapped ventricle to the subarachnoid space through a fenestration toward the basal cisterns. The patient was positioned supine on the operating table, with the head supported in a horseshoe headrest and rotated contra laterally. Pressure areas were carefully padded, and anatomical landmarks were verified using the navigation system. A 2 cm skin incision was made anterior to the tragus, and a burr hole was created. A trocar with an endoscopic sheath was inserted into the trapped ventricle, and the sheath was secured to a supporting arm fixed to the surgical table on the ipsilateral side. The trocar was then replaced with the endoscope, establishing a transcortical, transventricular surgical corridor. The choroid plexus was coagulated, and arachnoid webs encountered along the path were fenestrated through a surgical opening of the choroidal fissure, guiding access to the basal cisterns (Figure 3A–F). Upon advancing, the Liliequist membrane and basilar artery branches were visualized, and the endoscope tip was carefully introduced across the midline under direct endoscopic vision (Figure 3A–F).

Anatomical landmarks on the medial wall of the temporal horn were clearly visualized through the endoscope, and locations were confirmed with the navigation system, including the hippocampus, amygdala, and surrounding neurovascular structures within the basal cisterns. These structures included the anterior choroidal artery (AChA) and optic tract superiorly, as well as the posterior communicating artery (PCoA) and third cranial nerve inferiorly. Given the proximity to critical structures, the approach carries a risk of significant morbidity (1, 11).

**Figure 4:**
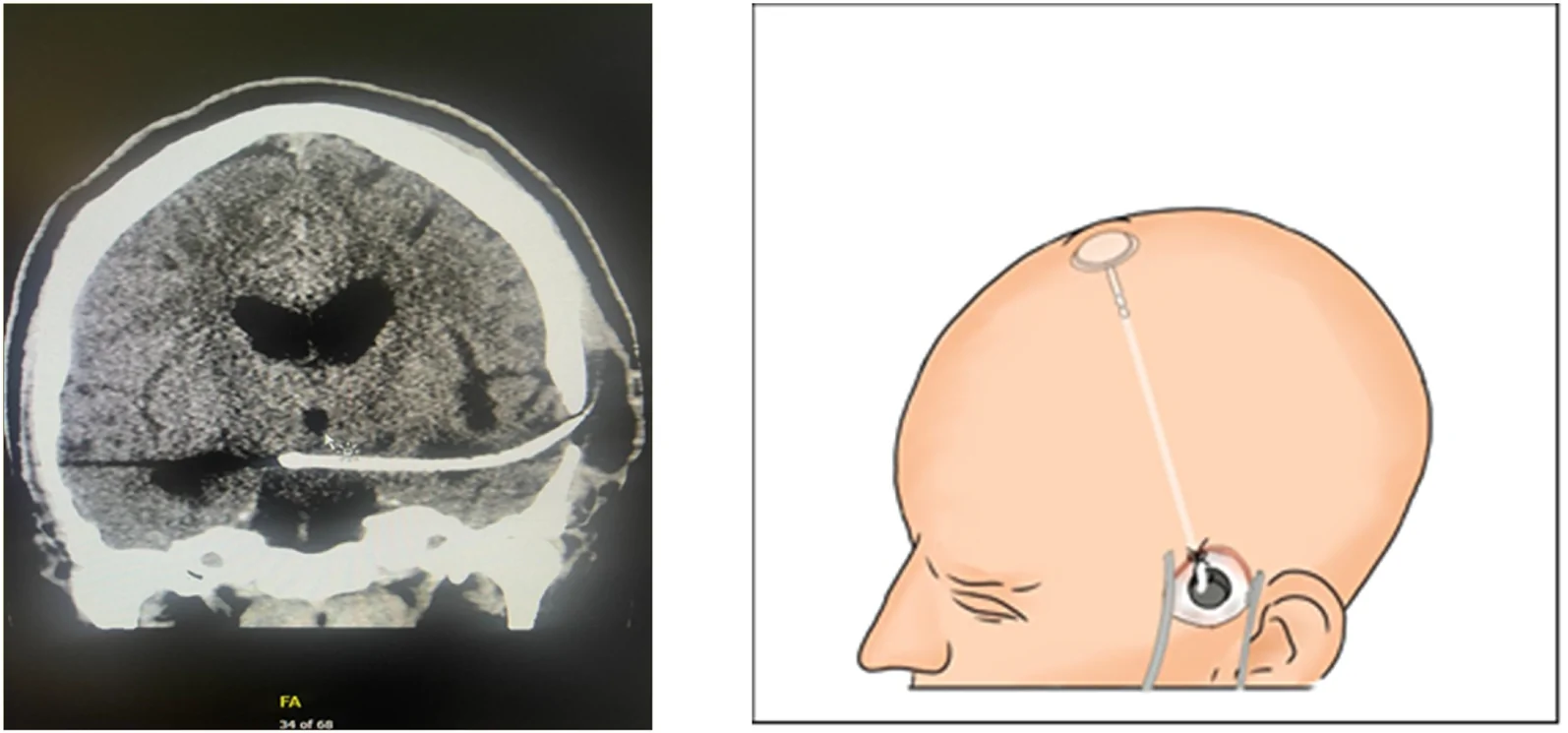
Postoperative CT brain, coronal view, following repositioning of the Ommaya reservoir catheter with additional fenestrations along the catheter tract. The image demonstrates no recurrence of the cyst, although a subsequent subcutaneous cerebrospinal fluid collection is noted. The illustration on the right shows the catheter fixation technique at the exit site, designed to prevent catheter displacement or migration.

The thinned medial wall was gently fenestrated using a blunt-tip dissector under meticulous dissection. Upon reaching the basal cistern, the stoma was dilated using a 4F Fogarty balloon catheter. An Ommaya reservoir was then inserted after creating multiple holes at the proximal end and along its track to facilitate drainage and to serve as a safety valve. The catheter hole was positioned near the cortex to ensure effective cerebrospinal fluid (CSF) washout from the trapped temporal horn into the cistern. Finally, the Ommaya reservoir was secured with silk sutures and fixed to the subcutaneous tissue. During the follow-up period from last revision for now 4 years, the patient's condition remained stable with no seizure. The follow up scans showed no recurrence, catheter migration or subcutaneous collection with complete resolution of trapped cyst (Figure 4).

## Discussion

Intraventricular tumors involving the temporal horn are considered a rare neoplasm. Their presentation varies depending on the tumor's size and location. Clinical manifestation can include; seizures, headaches, cognitive changes, and focal neurological deficits. Common types of intraventricular tumors include ependymomas, choroid plexus papilloma, and, less frequently germ cell tumors (9).

Entrapment of the temporal horn is a rare form of focal, non-communicating hydrocephalus. A standardized treatment protocol for this condition has not yet been established, and only a few cases have been reported in the literature (10). Various neuroendoscopic techniques for the resection of intraventricular tumors have been described, differing across studies based on surgeon preference, tumor morphology, and patient anatomy.

The concept of cisternal drainage, also known as cisternostomy, involves creating a fenestration into the subarachnoid cisterns to provide an alternative pathway for cerebrospinal fluid (CSF) circulation outside of the ventricles. This technique can help reduce the need for ventriculoperitoneal (VP) shunt placement. Cisternal drainage has been applied in cases of delayed hydrocephalus following aneurysmal subarachnoid hemorrhage (aSAH) (12) and traumatic brain injuries (13).

Our goal was to restore CSF flow from the cystic cavity (trapped temporal horn) to the basal cisterns, while using an Ommaya reservoir as a safety valve to regulate excess fluid accumulation and maintain a balance of CSF pressure between the cyst and the cisternal spaces.

Following the initial surgery, the drainage catheter had migrated further toward the cistern, leaving all the drainage holes within the cisternal space. To address this, we recreated multiple holes along the catheter track up to the cortex and tightly secured the catheter at the burr hole to prevent further migration.

When the patient returned few weeks later with a subcutaneous fluid collection, an initial attempt was made to manage the collection with a crepe bandage. However, the bandage was found to be loose. Therefore, we implemented a technique involving circumferentially stitched dressing gauze, tightly secured with silk sutures, to seal the subcutaneous space and prevent the development of a CSF collection (pseudo-meningocele).

The persistence of the subcutaneous collection may have been related to stagnation of high-protein CSF within the subcutaneous tissues and muscle layers, limiting absorption. Furthermore, an inadequately secured bandage could shift with head movement during sleep, reducing its efficacy. By tightly securing the dressing with sutures, we aimed to minimize the risk of requiring VP shunt placement and avoid the need for revision surgery.

Our underlying concept emphasizes the preference for Ommaya reservoir use over ventriculoperitoneal shunting, addressing complications as they arise. The use of a VP shunt carries risks of over-drainage or cyst collapse, potentially leading to obstruction of the catheter by the choroid plexus. In contrast, an Ommaya reservoir facilitates drainage driven primarily by the pressure gradient from the cyst to the intracranial compartment, promoting a safer and more physiologic flow. A comparative institutional review by Willis et al. analyzed outcomes of surgical treatment for posthemorrhagic hydrocephalus in 32 premature infants, of whom 15 underwent initial ventricular reservoir placement and 17 received ventriculoperitoneal (VP) shunts. Infants in the VP shunt group were significantly older and heavier at the time of surgery. Shunt revisions were required in 42% of the reservoir group and 53% of the VP shunt group, with the revision rate per patient significantly lower among those initially managed with a reservoir. No patient in the reservoir group required more than two revisions. Shunt infections occurred in 10.3% of patients, while two infants in the reservoir group died due to non-neurological complications related to prematurity. The authors concluded that low-birth-weight preterm neonates may benefit from initial CSF diversion with a reservoir followed by permanent shunting, as this approach was associated with fewer shunt revisions and improved management outcomes (14).

A variety of surgical strategies have been described for the management of trapped temporal horn, ranging from shunt placement to endoscopic fenestration and stenting. As summarized in Table 1, early endoscopic techniques demonstrated feasibility in achieving shunt independence (15–17), while subsequent reports highlighted their application in tumor-related entrapment and postoperative cases (18–20). More recent comparative series have shown that although shunting remains effective, it carries higher revision and infection rates compared with endoscopic alternatives (21). Together, these studies underscore the progressive refinement of minimally invasive approaches and support the rationale for incorporating endoscopic ventriculocisternostomy in selected cases such as ours.

**Table 1:** Clinical summary of case reported.

Author (Year, Journal)	Population/Age	Diagnosis	Intervention	Outcome/Follow-up
Schroeder & Gaab (2007, J Neurosurg) (16)	Adult case	Trapped temporal horn	Endoscopically guided fenestration of the choroidal fissure	Successful CSF diversion; shunt avoided; no recurrence reported. Neuroendoscopic techniques effectively treat obstructive hydrocephalus by restoring the blocked cerebrospinal fluid pathway or creating a bypass into the ventricles or subarachnoid spaces.
Hervey-Jumper SL (2010, J Neurosurg) (17)	3 Case who presented with focal compressive symptoms caused by temporal horn dilation.	Temporal horn entrapment	Frontal-to-temporal horn shunt	Effective temporal horn decompression with symptom resolution observed after up to 20 months of follow-up (at least 2 months in one patient who died from tumour progression).
Krähenbühl AK et al. (2013, J Neurosurg Pediatr) (18)	4 Pediatric patients 3 post-tumour resections. 1 post-meningitic multiloculated hydrocephalus	Trapped temporal horns	Endoscopic temporal ventriculocisternostomy	Safe and effective; sustained decompression; shunt-free follow-up. In two patients, a recurrent trapped temporal horn emerged. Reinfection was successfully treated in one case, while dilation of the trigone region followed by a septostomy was effective in the other. One patient developed postoperative meningitis, which was managed with appropriate treatment antibiotics.
Hana T et al. (2015, World Neurosurg) (19)	Post-glioblastoma resection	Temporal horn entrapment	Endoscopic ventriculocisternostomy + stent	Restored CSF circulation; no reaccumulation during follow-up. Combining ventriculostomy with stent placement may help reduce the risk of Trapped temporal hydrocephalous recurrence after endoscopic ventriculocisternostomy.
Paredes I, et al. (2017, J Neurosurg, case series (11)	4 cases (3 adults and 1 child)	1 Temporal AVM, 3 tumour cases	Endoscopic temporal ventriculocisternostomy	There were no procedure-related complications, and the decompression was successful, with patients remaining free of shunts throughout follow-up (4–24 months).
Lin Z, Wang et al. (2020, J Neurosurg) (20)	Clinical characteristics and treatment protocol for trapped temporal horn	Tumor-related trapped temporal horn (Trigone meningioma)	Endoscopic ventriculocisternostomy (protocol-based)	Improved outcomes with tailored management; validated in tumor-related entrapment.
Chen, Freeman & Warnke (2021, Interdiscip Neurosurg, (15)	6 cases; Meningioma (2). Renal cell Carcinoma. Low-grade glioma. Melanoma Breast Cancer	Atrial tumour with trapped temporal horn (either primary or secondary)	Stereotactic or endoscopic ventriculocisternostomy (various routes) with a Rickham catheter	With a median follow-up of 22.1 ± 7.8 months, all patients experienced symptomatic improvement, and the size of the temporal horn was normalized. No morbidity was reported.
Yamamoto T et al. (2022, Neurosurg Rev) (21)	Four patients technical report	Trapped temporal horn	Novel endoscopic ventriculocisternostomy + stenting with acryl puncture needle	Demonstrated feasibility; shunt-free at follow-up. None of the patients experienced recurrence of trapped temporal horn during an average follow-up of 39.3 months.
Lin Z et al. (2023, Neurosurg Rev, retrospective cohort) (22)	24 patients 13 (54.2%) patients receiving TFHS and 11 (45.8%) receiving VPS.	Trapped temporal horn	Temporal-to-frontal horn shunt vs. ventriculoperitoneal shunt	TFHS is cosmetic, cost-effective, and completely free of overdrainage, with revision rates comparable to those of other methods VPS.

TFHS, temporal-to-frontal horn shunt; VPS, ventriculo-peritoneal shunt.

## Conclusion

In conclusion, although intraventricular tumors of the temporal horn are rare, they present significant clinical challenges due to their varied symptomatology, potential for severe complications, and the risk of temporal horn entrapment. Current therapeutic options, including cisternal drainage and neuroendoscopic techniques, emphasize the need for individualized strategies tailored to each patient's anatomy and tumor characteristics. Accurate identification of critical anatomical landmarks remains crucial to minimizing surgical risks, particularly given the close relationship to vital neurovascular structures.

A promising development in postoperative CSF management is the innovative application of the Ommaya reservoir as an alternative to traditional ventriculoperitoneal shunts. This approach provides a safer and more controlled means of CSF diversion, reducing the need for repeat interventions and addressing complications commonly associated with shunt systems.

Future studies should focus on standardizing treatment protocols for intraventricular tumors and further evaluating the long-term outcomes of Ommaya reservoir use. Such efforts will advance patient care, improve safety, and help reduce morbidity in the management of these complex cases.

## Data Availability

The original contributions presented in the study are included in the article/Supplementary Material, further inquiries can be directed to the corresponding author.

## Supplementary material

Supplementary Figure S1 Postoperative: CT brain without contrast after removal of the EVD demonstrating entrapped left temporal horn with cystic dilatation.

Supplementary Figure S2 Left: Immediate postoperative non-contrast CT brain demonstrating the catheter in place with adequate cerebrospinal fluid (CSF) drainage. Right: Delayed postoperative non-contrast CT brain showing migration of the catheter toward the basal cistern with subsequent re-expansion of the cyst (indicated by the yellow arrow).
