# Supplementary material for: Case Report: Endoscopic-assisted cistern ostomy with Ommaya reservoir insertion for trapped temporal horn: surgical technique and case illustration
Source: Front Surg. 2026 Aug 6;13:1621466. doi: 10.3389/fsurg.2026.1621466 (PMC13490062; doi:10.3389/fsurg.2026.1621466)
Supplement: Supplementary file 1 [file Image1.pdf]

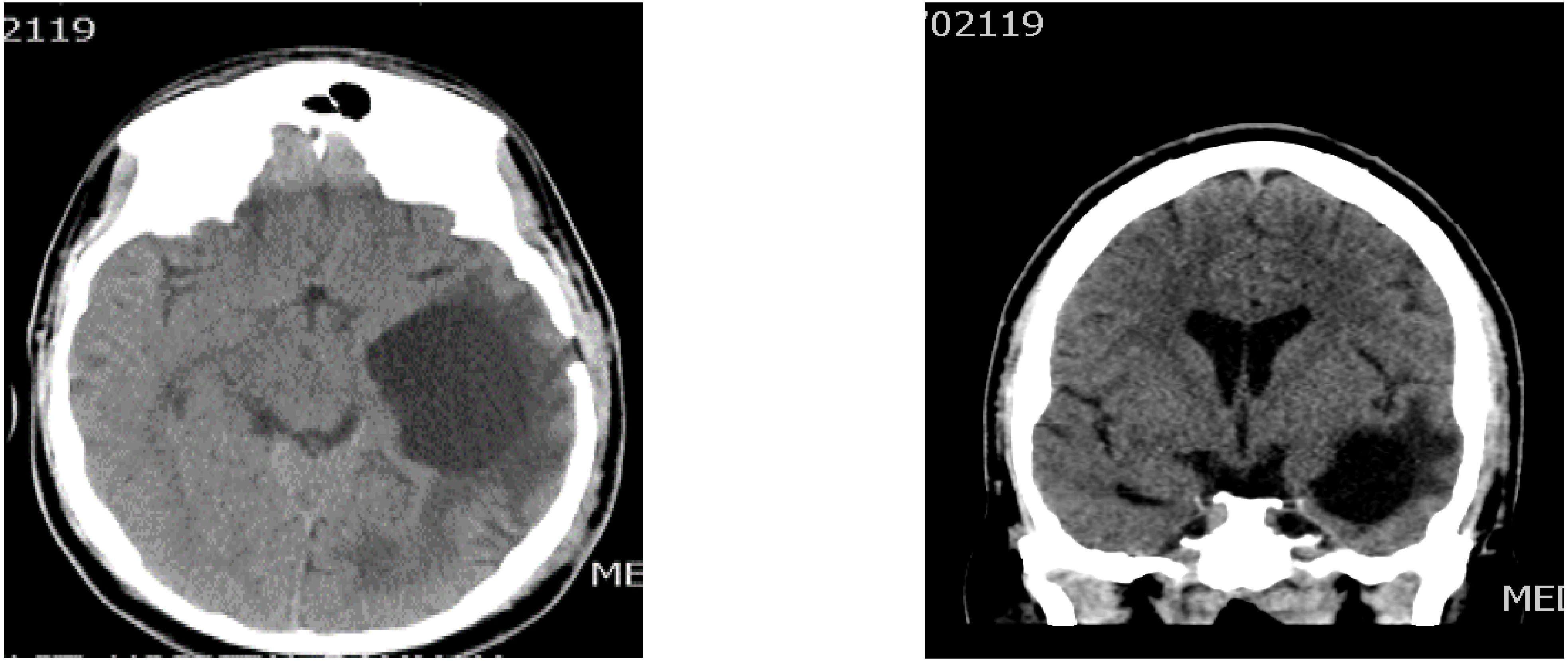

Supplementary FIGURE 1: Postoperative: CT brain without contrast after removal of the EVD demonstrating entrapped left temporal horn with cystic dilatation.
