# Supplementary material for: Case Report: Endoscopic-assisted cistern ostomy with Ommaya reservoir insertion for trapped temporal horn: surgical technique and case illustration
Source: Front Surg. 2026 Aug 6;13:1621466. doi: 10.3389/fsurg.2026.1621466 (PMC13490062; doi:10.3389/fsurg.2026.1621466)
Supplement: Supplementary file 2 [file Image2.pdf]

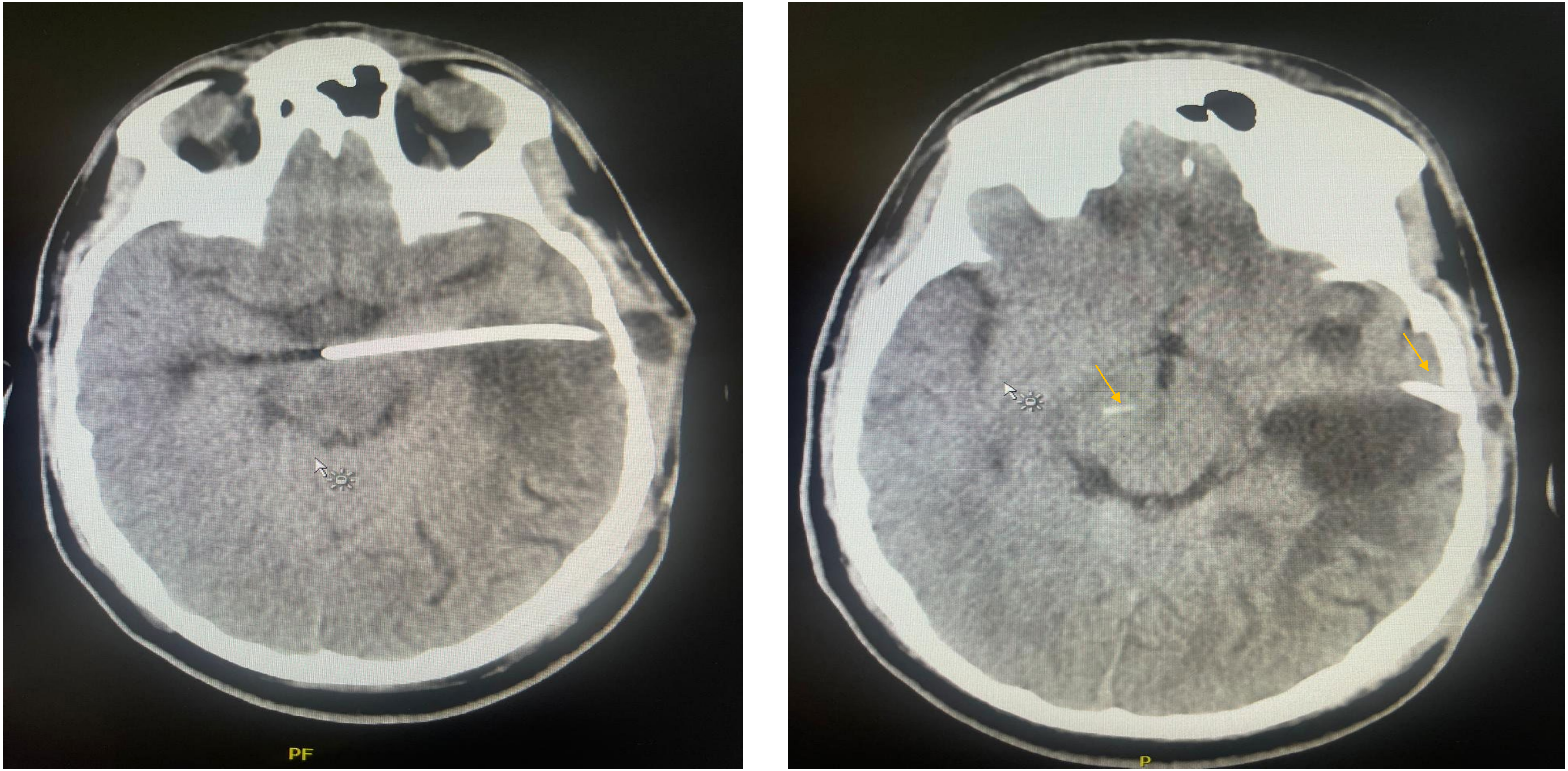


Supplementary Figure 2: Left: Immediate postoperative non-contrast CT brain demonstrating the catheter in place with adequate cerebrospinal fluid (CSF) drainage. Right: Delayed postoperative non-contrast CT brain showing migration of the catheter toward the basal cistern with subsequent re-expansion of the cyst (indicated by the yellow arrow).
